# Fibrous Disease of the Breast Can Obscure Breast Cancer Margins on Ultrasound: A Case Report

**DOI:** 10.7759/cureus.96630

**Published:** 2025-11-11

**Authors:** Kasumi Arizumi, Shoji Oura

**Affiliations:** 1 Department of Surgery, Kishiwada Tokushukai Hospital, Kishiwada, JPN

**Keywords:** breast cancer, diffusion-weighted images, enhanced posterior echoes, fibrous disease of the breast, no tumor margins

## Abstract

No studies have reported the occurrence of breast cancer within the fibrous disease of the breast to date. An 87-year-old nulliparous woman with a prior history of colon cancer surgery had been diagnosed with core needle biopsy-proven fibrous disease of the breast one year before. Follow-up mammography showed no change in the focal asymmetric density to which fibrous disease of the breast corresponded. Ultrasound also showed no marked changes in ill-defined low echoic areas, but clarified the disruption of the anterior margins of the mammary gland and more enhanced posterior echoes than before. Magnetic resonance imaging (MRI) of the target areas showed low signals on T1-weighted images, focal high signals in the large areas of low signals on fat-suppressed T2-weighted images, focal high signals on diffusion-weighted images, and focal both early and retained enhancement on dynamic studies, highly suggesting possible malignancy. Repeat core needle biopsy of the possible malignancy areas showed atypical cells with fibrous components, inflammatory cell infiltration, and vasculitis-like findings, leading to the diagnosis of breast cancer in the fibrous disease of the breast. Immunostaining showed estrogen and progesterone receptor negativity, human epidermal growth factor receptor type 2 positivity, and a high Ki-67 labeling index of 80%. The patient’s preference and her old age made us treat the patient not with neoadjuvant chemotherapy but with primary breast-conserving surgery and sentinel node biopsy. Pathological study showed no metastasis in the sentinel node and invasive breast cancer measuring 19 mm in the fibrous disease of the breast. The patient recovered uneventfully, was discharged on the second day after surgery, received one year of trastuzumab monotherapy as the postoperative adjuvant therapy due to the patient’s strong preference, and has been well for 14 months. Diagnostic physicians should note that unilateral fibrous disease of the breast should be evaluated with MRI before tissue sampling, and the change of posterior echoes is an important diagnostic clue for the diagnosis of breast cancer in the fibrous disease of the breast.

## Introduction

Fibrous disease of the breast is a benign disorder that has scattered mammary ducts and atrophic lobules in the abundant fibrotic stroma. This disorder is often observed in patients with diabetes, i.e., so-called diabetic mastopathy [1,2], but can be seen in patients without diabetes [3]. Fibrous disease of the breast is generally found in both breasts, but may also be detected in only one breast, often leading to a difficult diagnosis of this disorder for diagnostic physicians. Nulliparous women generally have dense breasts and often pose diagnostic difficulty using mammography when fibrous disease occurs in their unilateral breast. Although diabetes is a well-known risk factor for breast cancer [4], no studies have reported the occurrence of breast cancer within the fibrous disease of the breast to date. Why breast cancer occurrence within the fibrous disease of the breast is rare naturally remains uncertain. Background hyalinized stroma may be an unfavorable environment for breast cancer development.

It is well known that fibrous components not only give masses their hardness but also indistinct margins, when present at the mass borders mixed with tumor cells, on various images [5,6]. In addition, abundant fibrous components can weaken ultrasound waves and make the posterior echoes attenuated, often observed in scirrhous-type invasive ductal carcinomas. Therefore, breast cancer, when observed within the fibrous disease of the breast, can give physicians diagnostic difficulties. We herein report an extremely rare case of breast cancer within fibrous disease of the breast, in which fibrous disease of the breast obscured breast cancer margins on ultrasound and led to the breast cancer being diagnosed late.

## Case presentation

**Figure 1:**
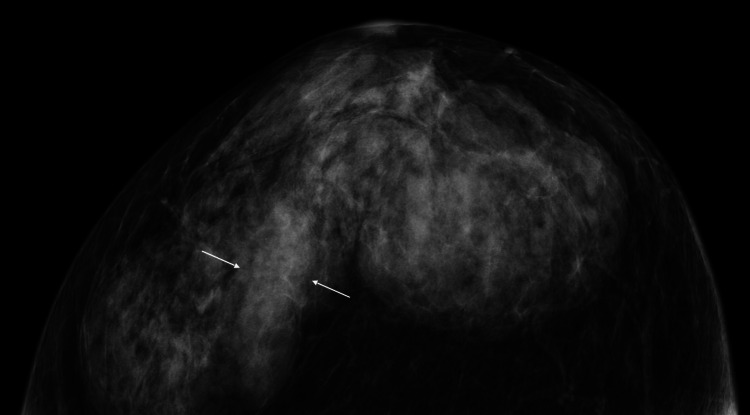
Mammography findings Cranio-caudal view mammography only showed focal asymmetric density (arrows) in the right dense breast.

An 87-year-old nulliparous woman with a prior history of colon cancer surgery just five years before, no diabetes, and no breast symptoms was referred to our hospital due to the right breast mass detected on follow-up computed tomography for colon cancer. Mammography only showed focal asymmetrical density in her right dense breast (Figure 1).

**Figure 2:**
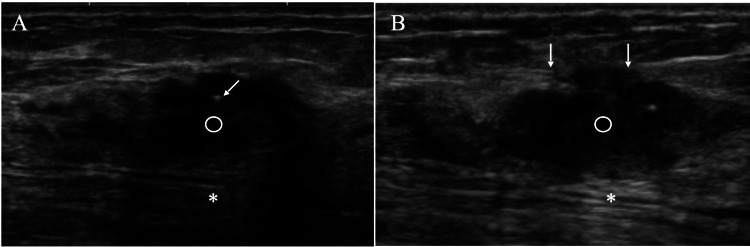
Ultrasound findings A. Ultrasound showed ill-defined low echoic areas (open circle) with an echogenic spot (arrow) and slightly attenuated posterior echoes (asterisk). B. Ultrasound showed no increase in size of the low echoic areas, disruption of the anterior margins of the mammary gland (arrows), and enhanced posterior echoes (asterisk).

Ultrasound showed ill-defined low echo areas with echogenic spots and slightly attenuated posterior echoes (Figure 2).

**Figure 3:**
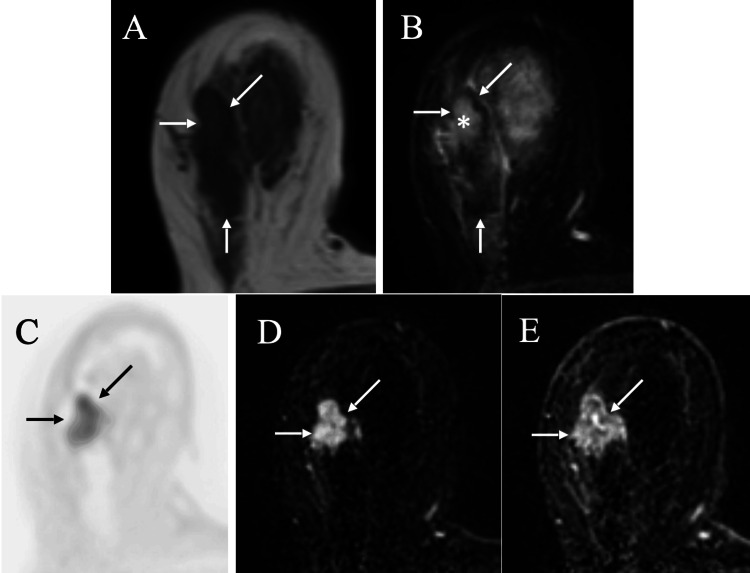
Magnetic resonance imaging (MRI) findings MRI of the target lesion showed low signals (arrows) on T1-weighted images (A), focal high signals (asterisk) in the large low signals (arrows) on fat-suppressed T2-weighted images, focal high signals (arrows) on diffusion-weighted images (C), and focal both early (D, arrows) and retained (E, arrows) enhancement on dynamic studies.

Under the judgment of possible malignancy, core needle biopsy was performed on the low echoic areas and pathologically showed fibrous components with peri-ductal lymphatic infiltration, leading to the diagnosis of fibrous disease of the breast. Follow-up mammography taken one year later still only showed a similar focal asymmetric density. Ultrasound also showed similar low-echoic areas to those observed one year before, but revealed the disruption of the anterior margins of the mammary gland and enhanced posterior echoes, prompting further detailed investigations. Magnetic resonance imaging (MRI) of the target areas showed low signals on T1-weighted images, focal high signals in the large low signals on T2-weighted images, focal high signals on diffusion-weighted images, and focal both early and retained enhancement on dynamic studies, highly suggesting possible malignancy (Figure 3).

**Figure 4:**
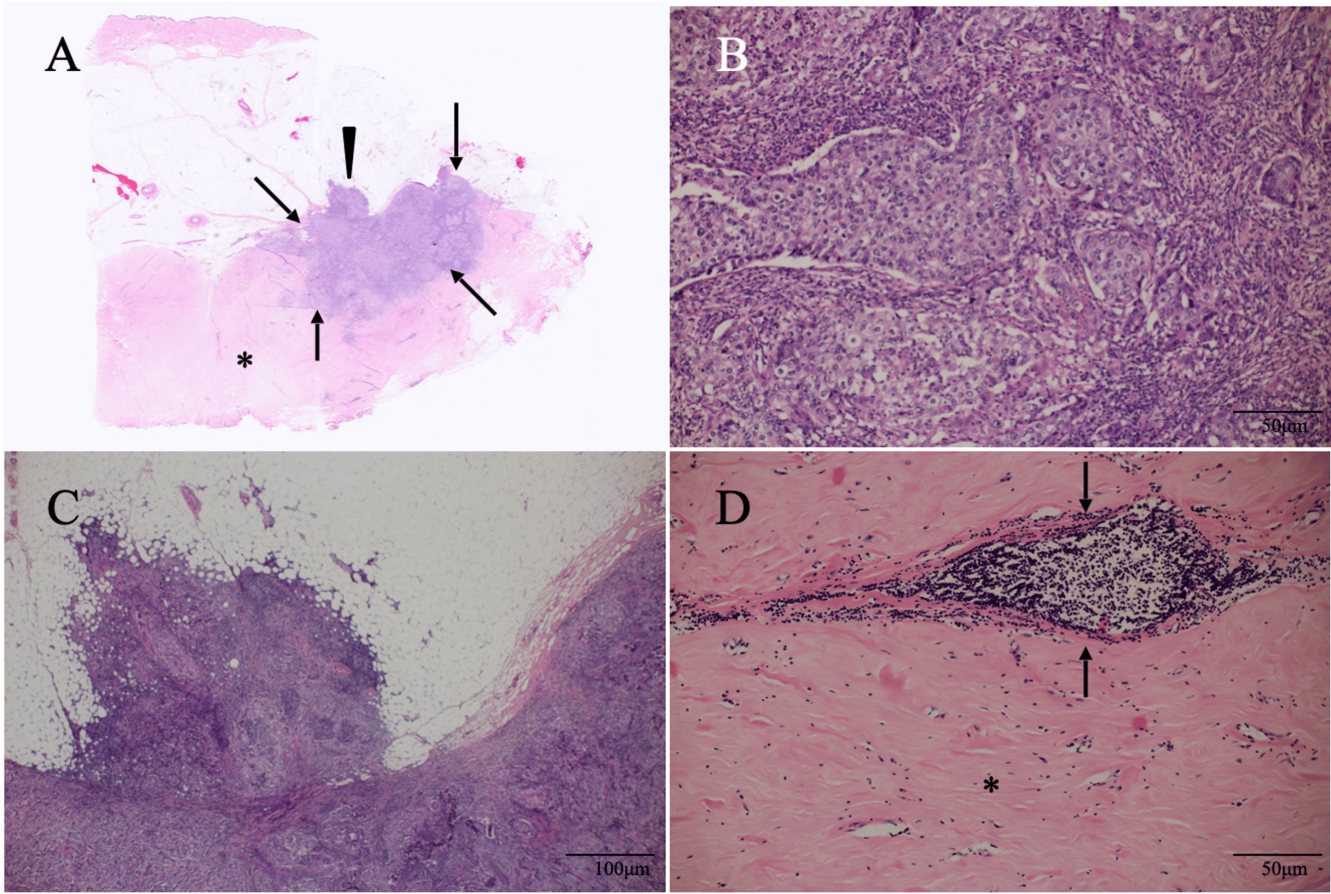
Pathological findings A. Low magnified view showed an irregular mass (arrows) with fat invasion (arrowhead) in the fibrous disease (asterisk). B. Magnified view showed atypical cells growing in an expansive fashion. C. Magnified view showed the fat invasion areas, well corresponding to the disruption of the anterior margins of the mammary gland on ultrasound. D. Magnified view showed abundant collagen fibers (asterisk) and peri-ductal inflammatory cell infiltration (arrows).

Repeat core needle biopsy of possible malignancy areas showed atypical cells with fibrous components, inflammatory cell infiltration, and vasculitis-like findings and led to the diagnosis of breast cancer in the fibrous disease of the breast. Immunostaining showed estrogen and progesterone receptor negativity, human epidermal growth factor receptor type 2 positivity, and a high Ki-67 labeling index of 80%. Patient’s preference and her old age, however, made us treat the patient not with neoadjuvant chemotherapy but with primary breast-conserving surgery and sentinel node biopsy. Pathological study clarified no metastasis in the sentinel node and invasive breast cancer measuring 19 mm in the fibrous disease of the breast (Figure 4).

The patient recovered uneventfully, was discharged on the second day after surgery, received one year of trastuzumab monotherapy as the postoperative adjuvant therapy due to the patient’s strong preference, and has been well for 14 months.

## Discussion

Although the patient was very old, 87 years, she had dense breasts presumably due to her nulliparity. Dense breasts made it difficult for us to determine whether the fibrous disease of the breast, including breast cancer, was malignant or not. Despite no remarkable changes, both focal asymmetric density on mammography and low echoic areas on ultrasound, ultrasound revealed disruption of the anterior margins of the mammary gland and more enhanced posterior echoes one year later.

On ultrasound, mass shapes are formed by ultrasound wave reflection, which requires interfaces much larger than the ultrasound wavelength [5,6]. In addition, the two pathological components forming the interfaces must have different acoustic impedances in order to clarify the mass shape on ultrasound. We were unable to directly measure the difference in acoustic impedance between cancer cells and fibrous components in this case, but we think that the difference was presumably not very large. Cancer cells pathologically proliferated densely in an expansive manner in this case without forming papillary/tubular structures and fibrous disease of the breast consisted of abundant fibrous components lacking micro voids often seen in fibrous component-bearing disorders [7] such as scirrhous type invasive ductal carcinomas and sclerosing adenosis, possibly leading to no mass shape formation of the breast cancer at least lateral and posterior margins due presumably to little difference in acoustic impedance between cancer cells and fibrous disease of the breast consisting components [8].

It is well known that the presence of abundant fibrous components in the mass can attenuate the posterior echoes [9]. Posterior echoes, however, changed from slightly attenuated to enhanced in one year in this case, suggesting the increased proportion of cancer cells within the ill-defined low echo areas. Comparison between the pathological and ultrasound findings naturally leads to the fact that ill-defined low echoes consisted of fibrous disease of the breast, including breast cancer. We, therefore, can easily speculate that the initial pathological negative study was due to the tissue sampling not to the breast cancer areas but to the fibrous disease of the breast areas encompassing the breast cancer.

Fibrous disease of the breast generally affects both breasts, but can have its lesion only in one breast. Diagnostic physicians, therefore, can avoid underestimation of the unilateral target lesion with the MRI evaluation when suspected of possible fibrous disease of the breast. MRI evaluation can not only clarify breast cancer in the fibrous disease of the breast but also lead to the omission of unnecessary core needle biopsy in the benign disorder of fibrous disease of the breast. In addition, diffusion-weighted images further help diagnostic physicians in diagnosing patients, even with renal impairment, an important diagnostic clue for the diagnosis of breast cancer in the fibrous disease of the breast [10]. Changes of posterior echoes also seem to be an important finding suggestive of breast cancer within the fibrous disease of the breast.

## Conclusions

Diagnostic physicians should evaluate the presumed unilateral fibrous disease of the breast with contrast-enhanced MRI on deciding whether to apply needle biopsy to the target lesion or not. In addition, for patients with severe renal impairment, diffusion-weighted images can provide important image findings about the presumed breast cancer in the fibrous disease of the breast. Changes of posterior echoes can also be an important diagnostic clue for the diagnosis of breast cancer within the fibrous disease of the breast.
